# Influence of Cathode Origin on the Crystallization of LiNbO_3_ Coatings on Ni‐Rich Layered Oxides

**DOI:** 10.1002/cplu.70248

**Published:** 2026-09-16

**Authors:** Valeriu Mereacre, Yiran Guo, Ruochen Xu, Anna‐Lena Hansen, Michael Knapp, Helmut Ehrenberg, Joachim R. Binder

**Affiliations:** ^1^ Institute for Applied Materials – Energy Storage Systems Karlsruhe Institute of Technology Eggenstein‐Leopoldshafen Germany

**Keywords:** layered oxide cathodes, LiNbO_3_, lithium‐ion batteries, surface coating

## Abstract

Layered transition‐metal oxides are widely used as cathode materials for lithium‐ion batteries, and surface coating with lithium niobates is a common strategy to improve their electrochemical performance. In many studies, the formation of crystalline Li_3_NbO_4_ is reported as the final product of Li–Nb–O coating processes after thermal treatment. However, experimental observations obtained for different cathode morphologies and processing conditions indicate that the structural evolution of Li–Nb–O coatings is more complex. A systematic analysis of temperature‐dependent diffraction data, combined with comparison to literature results for structurally related systems, shows that Li_3_NbO_4_ is not the only crystalline phase that can form during high‐temperature treatment. Depending on coating optimization, cathode morphology, and thermal history, the formation of an Nb‐stabilized rock‐salt–type Li–Nb–M–O phase, described as *x*Li_3_NbO_4_−(1−*x*)LiMO_2_ (M = Ni, Co, Mn), is observed. These findings explain the absence of LiNbO_3_ and/or Li_3_NbO_4_ reflections in certain coated materials and demonstrate that the frequent assignment of Li–Nb–O coating products solely to Li_3_NbO_4_ is incomplete. The results were obtained from samples containing increased Li–Nb coating amounts to facilitate detection of crystalline reaction products by laboratory X‐ray diffraction (XRD).

## Graphical abstract

Li–Nb coatings evolve differently on different Ni‐rich cathodes. Depending on the host material and thermal treatment, LiNbO_3_, Li_3_NbO_4_, or Nb‐stabilized rock‐salt Li–Nb–M–O phases form, highlighting the importance of the cathode’s synthesis history for interpreting coating mechanisms.

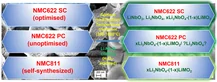

## 1 Introduction

There are many materials that can be used as cathode materials [1, 2]. For these materials to be effective, their properties often need to be optimized. To achieve an effective optimization, researchers must possess full information about cathode materials' structure and composition, including whether the materials are coated or doped, and if so, with which elements and in what quantities. If the materials are self‐synthesized in small quantities in the laboratory, this information is, of course, more easily available. However, when larger quantities are required and researchers seek to purchase these materials from different companies, such information is often not provided by manufacturers, who may be reluctant to share proprietary details. Commercial materials are often optimized for stability and performance before being made available for purchase. As a result, researchers frequently encounter difficulties when working with these substances due to the lack of available information. Knowing the precise structure and composition of these materials makes it easier to interpret the effects and results obtained from their study. To effectively address emerging problems and new findings or to explain the causes of negative results, researchers must have comprehensive information about these materials. Some time ago, we developed a coating method using H_2_O_2_ as an activating agent, which proved very successful for coating spinels with LNMO (LiNi_0.5_Mn_1.5_O_4_) [3, 4, 5]. The activation by H_2_O_2_ enhances the surface reactivity of the active material, which significantly reduces the reaction time and ensures the uniformity of the coating layer. We believed it could also work for layered oxides NMC (LiNi_*x*_Mn_*y*_Co*_z_* O_2_). However, during our attempts to obtain LiNbO_3_‐coated Ni‐rich layered oxides, we encountered several challenges while addressing various questions. Throughout our research in this field, we conducted numerous experiments but refrained from publishing the data because we were uncertain about their consistency. Using the same coating reagents, identical coating chemistry, thermal treatment, and molar quantities as those for the LNMO materials, we coated several commercial NMCs. However, the results were puzzling: both X‐ray studies and electrochemical analyses contradicted previously published results and our expectations. This prompted us to synthesize our own NMCs. The electrochemical performance of these materials (not shown in this work) demonstrated that the H_2_O_2_ coating method was effective, resulting in a significant improvement. However, the X‐ray diffraction (XRD) results contradicted findings in the literature. This discrepancy prompted us to analyze the results reported in various publications in greater detail, raising questions about reliability of some of the published data. In this paper, we will attempt to address those questions. We will examine sol–gel reactions in greater detail as they yield the most homogeneous coatings [6]. While coatings can also be produced using dry coating methods [7], these do not achieve the same level of homogeneity. Furthermore, we will investigate sol–gel reactions because our method involving H_2_O_2_ resembles a type of sol–gel reaction, even though it is not entirely accurate to refer to it as such since no gel is formed during the reaction. In this work, two isostructural commercial LiNi_0.6_Mn_0.2_Co_0.2_O_2_ cathode materials, a single‐crystalline (NMC622SC) and a single‐crystalline Ni82, a polycrystalline (NMC622PC), as well as a polycrystalline self‐synthesized NMC811 variant, were coated with Li–Nb oxides using identical synthesis and calcination protocols and investigated by laboratory and synchrotron X‐ray diffraction as well as scanning electron microscopy (SEM). While LiNbO_3_ and Li_3_NbO_4_ reflections are detected for the single‐crystalline material after calcination in the range of 450–800°C, the polycrystalline counterparts exhibit either strongly suppressed or absent Li–Nb–O reflections, despite uniform surface modification consistent with a homogeneous Li–Nb‐derived surface layer. The obtained results are compared with literature results for structurally related systems.

## 2 Experimental Section

### 2.1 Synthesis of Cathode Active Material

NMC622SC and Ni82 materials were purchased from MSE Supplies, and NMC622PC was purchased from BASF SE. The preparation of NMC811SS (self‐synthesized) involved several steps: initially, Ni_0.8_Mn_0.1_Co_0.1_(OH)_2_ powder was produced using a conventional coprecipitation technique in a continuously stirred tank reactor, following a procedure akin to that outlined by Van Bommel et al. [8]. Subsequently, this powder was combined with a predetermined quantity of Li_2_CO_3_ or LiOH and then heated at either 800 or 775°C in an oxygen atmosphere.

### 2.2 Surface Coating via a Wet H_2_O_2_ Chemistry Approach

The lithium niobium oxide‐based surface coating on NMC622SC, NMC622PC, NMC811SS, and Ni82 active materials was prepared using a chemical approach with H_2_O_2_ as an activating agent, as described in our earlier work [9]. The nominal LiNbO_3_ loading used for the structural investigations was 6 wt.% for NMC622SC/NMC622PC and 5 wt.% for NMC811SS/Ni82 samples**.** To prepare the Li/Nb coating solution, 0.30 g (0.94 mmol) of niobium(V) ethoxide (Aldrich) was dissolved in 20 mL of absolute ethanol, while 0.057 g (1.08 mmol) of lithium ethoxide (Aldrich) was separately dissolved in 40 mL of absolute ethanol. The two solutions were then combined, followed by the addition of 60 mL of hydrogen peroxide (30%) and ~0.1 mL (8 drops) of 65% nitric acid (HNO_3_). The resulting mixture was stirred until a clear solution was obtained, indicating complete dissolution of the precursors. 1.2 g of the respective cathode‐active material and 13 mL of absolute ethanol were added to a round‐bottom flask (*V* = 50 mL). The resulting suspension was stirred for approximately 3 min before a certain volume of Li/Nb coating solution (corresponding to ~5‍–‍6 wt.% LiNbO_3_) was added dropwise under continuous stirring. Stirring was continued until gas evolution ceased. The suspension was then decanted, and the powder was washed with ethanol, centrifuged, dried at 80°C, and calcined in oxygen first at 250°C for 2 h, followed by 350–1000°C for 5 h at a heating rate of 4°C/min^−1^. The samples were subsequently cooled to room temperature at a rate of 2°C/min^−1^. The selected temperatures were chosen to follow the crystallization sequence of Li–Nb‐containing phases from the amorphous state to LiNbO_3_, Li_3_NbO_4_, and finally rock‐salt‐type reaction phases.

### 2.3 Material Characterization

A scanning electron microscope SEM (Zeiss Supra 55) was used to analyze the morphology. Powder X‐ray diffraction was recorded using a Bruker D2 Phaser Diffractometer with Cu–*K*_α_ radiation (*λ* = 1.5406 Å) and a LynxEye XE‐T detector in reflection mode. XRD patterns were recorded over a 2*θ* range of 10°–80°/90° with step size of 0.02° and counting time of 1 or 2 s per step. Rietveld refinements were performed on TOPAS‐V6 (Bruker AXS) and software jEdit.

## 3 Results and Discussion

### 3.1 XRD and SEM Analysis of Li–Nb Oxide Coatings on Commercial NMC622

**FIGURE 1:**
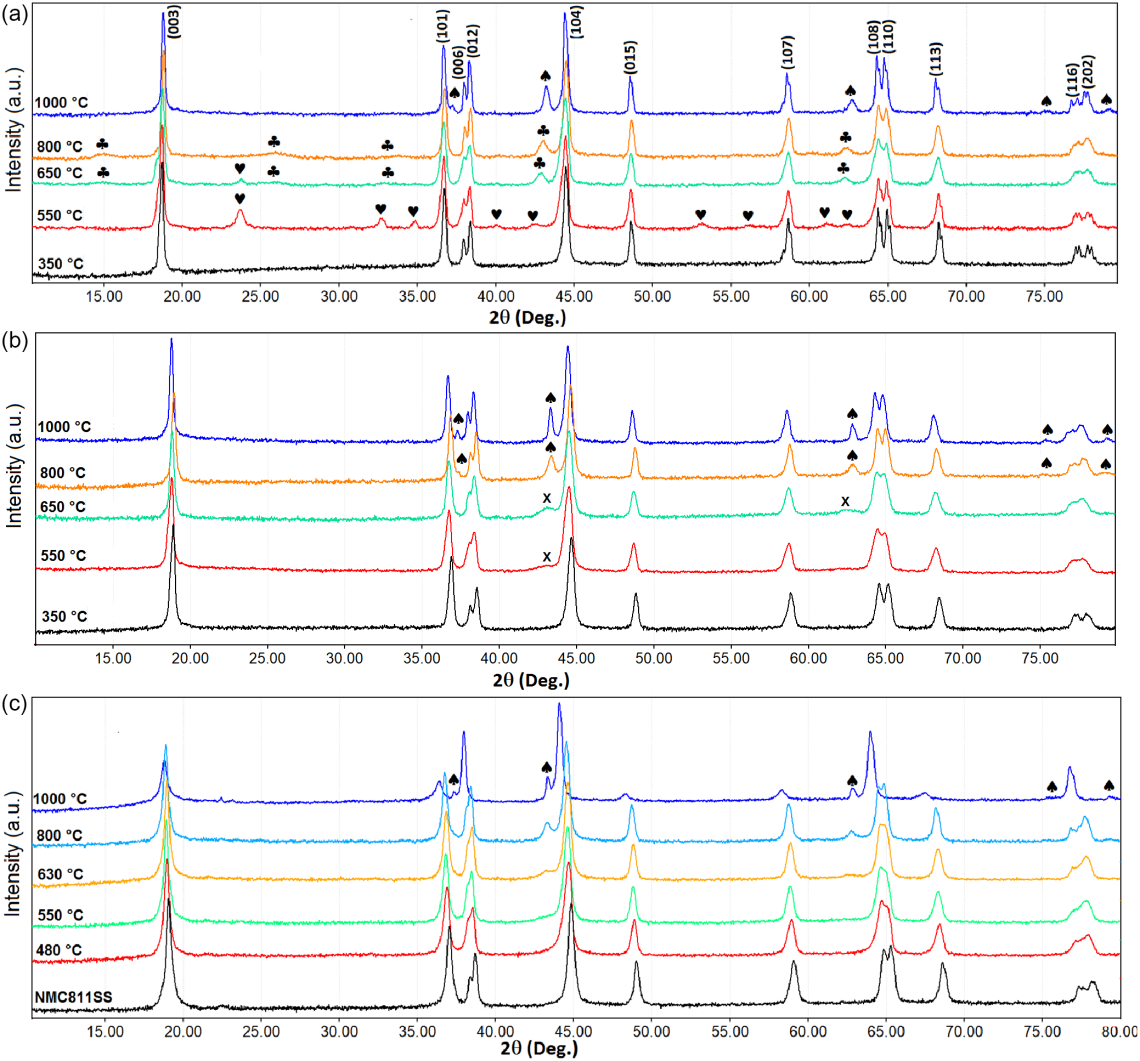
XRD for 6 wt.% Li–Nb oxide coated samples NMC622SC (a) and NMC622PC (b) heated at different temperatures: 350, 550, 650, 800, and 1000°C; **♥** is LiNbO_3_, **♣** is Li_3_NbO_4_, **♠** is *x*Li_3_NbO_4_−(1−*x*)LiMO_2_, and **x** is most probably Li_3_NbO_4_ or a mixture Li_3_NbO_4_/*x*Li_3_NbO_4_−(1−*x*)LiMO_2_. (c) XRD patterns of the 5 wt.% Li–Nb oxide‐coated NMC811SS sample heated to various temperatures: 480, 550, 630, 800, and 1000°C). The symbol ♠ indicates the presence of the *x*Li_3_NbO_4_−(1−*x*)LiMO_2_; the characteristic reflections at approximately 37°, 43°, 63°, 75°, and 79° correspond to the (111), (200), (202), (311), and (222) lattice planes, respectively. The presence of Li_3_NbO_4_ as a separate phase is not excluded.

**FIGURE 2:**
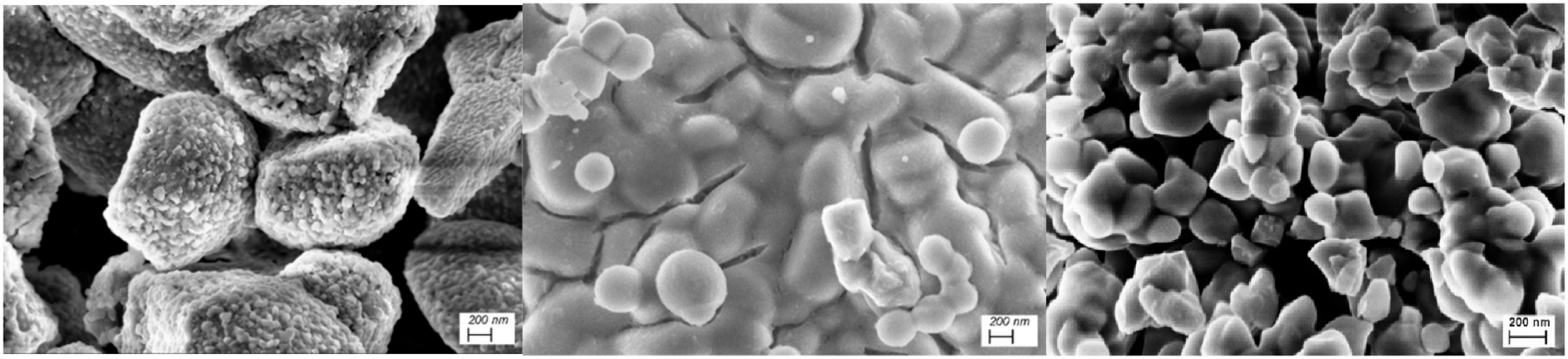
SEM images of the NMC622SC‐6%LN‐650 (left) and NMC622PC‐6%LN‐650 (center), both calcined at 650°C, and of the 5 wt.% Li–Nb oxide‐coated NMC811SS (right) sample calcined at 480°C.

First, the results will be shown, which prompted to consult the published findings in this field in more details. Two isostructural commercially available materials were coated with Li–Nb oxides: LiNi_0.6_Mn_0.2_Co_0.2_O_2_ (single crystal) from MSE Supplies (NMC622SC) and LiNi_0.6_Mn_0.2_Co_0.2_O_2_ (polycrystalline) from BASF SE (NMC622PC). Lithium and niobium ethoxides have been used for coating of both cathode materials using hydrogen peroxide as activating agent [3, 4, 5, 9]. This coating process has been investigated previously in our group, where scanning transmission electronic microscopy (STEM) analyses demonstrated the formation of a homogeneous Li–Nb–O coating with a thickness of only a few nanometers on NMC622SC after heat treatment at 350°C [9]. The present study therefore focuses on the crystallographic evolution of this previously established coating during subsequent annealing at higher temperatures rather than on its initial morphology. As usual, for the electrochemical studies, the cathode materials were coated with a 0.5 wt.% coating, while for other studies, including XRD, a 5–6 wt.% coating was used. A nominal coating loading of 5–6 wt.% was selected to enhance the detectability of Li–Nb‐containing crystalline reaction products by laboratory XRD. This higher loading was used exclusively for phase‐identification studies and should not be interpreted as the amount of crystalline Li–Nb oxide present after calcination. After the coating process, the samples were then calcined at different temperatures (350–800°C) to determine the crystallization temperature of the coating and its phases. What was interesting is that between 450 and 800°C, for the NMC622SC sample, the reflections for the Li–Nb‐oxides (LiNbO_3_ and Li_3_NbO_4_) were detected, but for the polycrystalline NMC622PC, no reflections or only some of them were detected. As a result, the provenance of the polycrystalline sample was verified. Also, the coating reactions were repeated, and to gain further insights into the changes in the surface environment, SEM measurements were also conducted. This additional test was performed to verify whether the surface was indeed covered by the coating and to assess any changes in its morphology. The X‐ray and SEM results are presented in Figures 1 and 2, respectively. The SEM images of the pristine, uncoated NMC622SC and NMC622PC are shown in Figure S1. The SEM images for the samples coated with 6 wt.% Li–Nb oxide and calcined at 650°C clearly indicate the presence of the coating on both of them (Figure 2). The images shown in Figure 2 are representative of the observed morphology; multiple regions of each sample were examined, and similar morphological features were observed across the different areas. For the polycrystalline sample, NMC622PC, it is also possible to roughly estimate the thickness of the coating, which appears to be relatively thick and homogeneous. This material was calcined again at the same temperatures (350–800°C) as the single‐crystal sample, NMC622SC, using the same gas, in the same oven, and at the same position within the oven. A logical question arises: why does the XRD not show any Li–Nb oxide reflections for the polycrystalline sample NMC622PC? SEM images for this sample undoubtedly show the presence of the coating. So, why are there no new reflections typical of LiNbO_3_ and Li_3_NbO_4_ oxides in the XRD results as observed in the case of NMC622SC? In the following sections, we will seek to answer this question by analyzing various publications that report different synthetic and coating approaches. The selected calcination temperatures were chosen to monitor the crystallization of the Li–Nb coating. The objective of this work was not to investigate the thermal evolution of the layered oxide host itself but to compare how identical Li–Nb coating procedures evolve on different cathode materials under identical heat treatment conditions.

### 3.2 Challenges With Commercial NMC Materials and the Need for Self‐Synthesized NMC811

The main target of a production company is to produce a material that is ready to be used in a battery and that delivers good stability, high performance, and stable capacity. Such materials are optimized and improved exactly for achieving such properties. Many of them are not prepared for further chemical or nonchemical treatment or additional research. As mentioned above, most manufacturers of cathode materials are reluctant to share proprietary details. Thus, we are confident in the bulk structure and its elements of these commercial materials but not in their chemical properties and their postsynthetic “history”: are they coated, which material was used for the coating, is this coating amorphous or crystalline, are they substituted and with which ions, which method was used for the synthesis, which calcination temperatures, how many times they were calcined, etc. All these conditions and information are very important for a researcher who would like to do extra research on such materials. Of course, this information is not always available. Therefore, we decided to synthesize our own compound‍—‍self‍‐‍synthesized NMC811 (LiNi0.8Mn0.1Co0.1O2)‍—‍(NMC811SS). This composition was selected because NMC811 is the predominant variant among extensively studied and published Li/Nb‐coated NMC materials. The synthesis was done using a standard procedure (see Experimental Part): Ni_0.8_Mn_0.1_Co_0.1_(OH)_2_ precursor was obtained using a coprecipitation process. It was then mixed with Li_2_CO_3_ (molar ratio, Li/M = 1.05/1) and calcined at 800°C for 5 h under O_2_ flow. The final material, NMC811SS, was then coated with 5 wt.% Li–Nb oxide (Figures 1 and 2) using the same method as for NMC622SC and NMC622PC. After calcination at different temperatures (480–800°C), as in case of NMC622PC, we encountered difficulties in identifying Li–Nb oxides using X‐ray techniques (Figure 1c). It is important to mention that an additional NMC811 was synthesized by mixing the Ni_0.8_Mn_0.1_Co_0.1_(OH)_2_ precursor with LiOH and calcined at 775°C under the same conditions. After being coated with 5 wt.% Li–Nb oxide, as with NMC622PC and NMC811SS, this material also shows no detectable reflections corresponding to crystalline LiNbO_3_ within the investigated temperature range, raising questions regarding the presence of reflections from the Li_3_NbO_4_ phase. The same qualitative trend was also observed for additional commercial cathode materials from different suppliers; however, the corresponding data are not presented here because their disclosure was restricted by the suppliers.

### 3.3 Formation of LiNbO_3_, Li_3_NbO_4_, and *x*Li_3_NbO_4_−(1−*x*)LiMO_2_ in Li–Nb Coated NMC Under High‐Temperature Treatment

Characteristic signals for the coated Li–Nb oxides have been observed in several reported sol–gel and solid‐state synthesis approaches for various isostructural layered oxides [10, 11, 12, 13, 14, 15]. Also, for the self‐synthesized spinel compounds, LiNi_0.5_Mn_1.5_O_4_, coated with 2.5 wt.% LiNbO_3,_ it is possible to detect crystalline LiNbO_3_ using XRD after thermal treatment at sufficiently high temperatures (800°C) [3].

The coexistence of crystalline LiNbO_3_ and Li_3_NbO_4_ phases in sol–gel‐derived Li–Nb oxide coatings has recently been confirmed. An illustrative example involving NMC811 coated with Li–Nb oxides was reported by Xin et al. [10]. The XRD reflections corresponding to the Li–Nb compounds were distinct and unambiguous, supporting the authenticity of the reported results. A similar temperature‐dependent evolution of XRD reflections was observed in our study of commercial NMC622SC (Figure 1a). Like NMC622SC, NMC811 reported by Xin et al. (NMC811X) is a commercial one from Ecopro. Because the detailed synthesis and postsynthesis treatment of the commercial material were not reported, its surface state and degree of industrial optimization cannot be established from the available information. Nevertheless, the material exhibits a coating response distinct from that observed for our self‐synthesized NMC811SS. As we mentioned before, usually, the producer does not provide detailed information about the postsynthesis treatment. Assuming the surface was already optimized, coating with Li/Nb compounds followed by calcination at various temperatures leads to the crystallization of different phases. LiNbO_3_ is primarily formed at temperatures between 400 and 600°C, whereas Li_3_NbO_4_ appears at 700–800°C. At higher temperatures, a partial reaction between the Li/Nb coating and the active material occurs, resulting in partial substitution of NMC with Nb ions, which likely occupy transition‐metal sites, displacing some of the original cations [10]. Unfortunately, measurements at higher temperatures are not provided; therefore, it is not possible to judge about the products that may be formed. We have done such experiments: all samples, NMC622SC, NMC622PC, and NMC811SS, have been calcined at additional temperatures: 900 and 1000°C (Figure 1a–c). At 900°C (not shown because it is similar to 800°C) for NMC622SC, three broad reflections typical for Li_3_NbO_4_ are still visible at 2*θ* ≈ 15.0° and in the range between 2*θ* ≈ 25.0° and 35.0°; then, at 1000°C, they are not any more noticeable (Figure 1a). But a clear new weak reflection appeared at 2*θ* ≈ 37.1°, and additional broad and weak reflections at 2*θ* ≈ 75.1° and 79.1°. Based on the reflection features, we assume that in NMC622SC, there is a transformation from Li_3_NbO_4_ to a new, third material, a cation‐disordered rock‐salt phase, *x*Li_3_NbO_4_−(1−*x*)LiMO_2_ [16], as indicated by the refinement results based on the diffraction patterns (Figure S2c,d). However, due to the high similarity between these phases (both having cubic symmetry), neither the exact formation temperature nor the precise value of *x* in this rock‐salt phase can be accurately determined. Depending on their value, various compositions may form between ~800 and 1000°C, resulting in small shifts in the XRD reflections due to changes in the lattice parameters [17]. Because XRD is a bulk‐sensitive technique, the detected Li–Nb‐containing reflections cannot be assigned exclusively to phases located within the surface coating. Contributions from Li–Nb‐containing phases formed at the interface or distributed within the cathode particles cannot be excluded.

**FIGURE 3:**
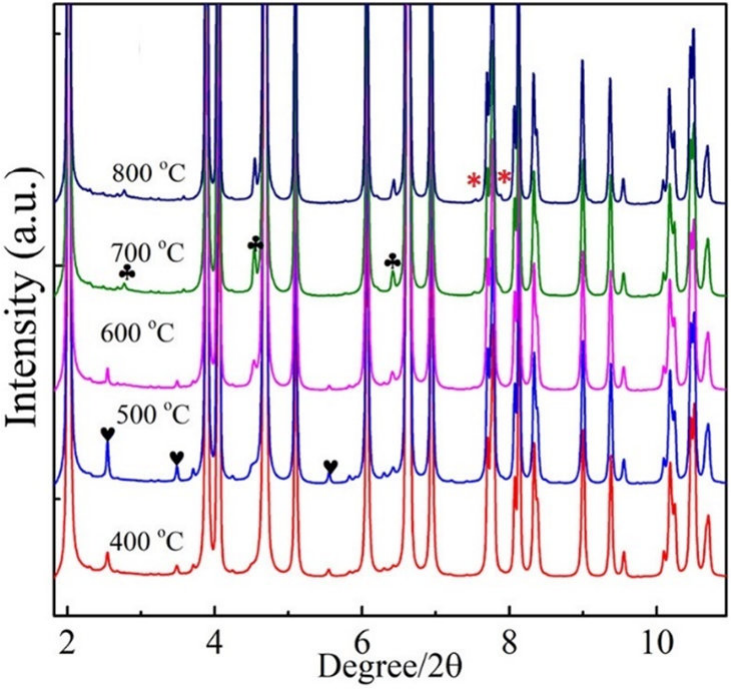
Synchrotron XRD patterns of 1.4% Nb‐modified NMC811X heated at different temperatures; **♥** is LiNbO_3_, and **♣** is Li_3_NbO_4_. The two reflections at 2*θ* ≈ 7.6° and 7.9° marked with **∗** can be attributed to the (311) and (222) planes of the *x*Li_3_NbO_4_−(1−*x*)LiMO_2_ phase. Reprinted with permission from Ref. [10]. Copyright 2021 American Chemical Society.

In general, we believe that the rock‐salt phase *x*Li_3_NbO_4_−(1−*x*)LiMO_2_ may begin to form in the NMC622SC sample already at approximately 650–800°C. This assumption is supported by findings reported by Xin et al., who observed the formation of a rock‐salt structure in NMC811X at 700°C using high‐resolution transmission electron microscopy (HR‐TEM) combined with fast Fourier transform (FFT) diffraction analysis [10]. However, the appearance of this phase was only briefly discussed in their study. Notably, two additional reflections at 2*θ* ≈ 7.6° and 7.9° (synchrotron radiation, *λ *= 0.18266 Å), which can be attributed to the (311) and (222) planes of the *x*Li_3_NbO_4_−(1−*x*)LiMO_2_ phase (Figure 3), are also clearly visible in the synchrotron XRD patterns reported by Xin et al. [10], further supporting the presence of the rock‐salt phase *x*Li_3_NbO_4_−(1−*x*)LiMO_2_ in NMC811X.

In the case of the NMC622PC and NMC811SS materials, which are not optimized, the active substances more readily react with Li–Nb compounds. Between 550 and 1000°C, the formation of crystalline *x*Li_3_NbO_4_−(1−*x*)LiMO_2_ (see XRD of NMC622PC at 800 and 1000°C, Figure 1b and the simulated patterns after Rietveld refinement, Figure S2e,f) likely occurs without the detectable formation of intermediate crystalline LiNbO_3_ or Li_3_NbO_4_ phases, as confirmed by XRD analysis. (also, synchrotron XRD for NMC622PC at 800°C confirms this (Figure S3)). However, their presence cannot be entirely excluded. It is more likely that these intermediate phases exist in an amorphous or nanocrystalline form, which would require more advanced characterization techniques to be detected. Recently, crystalline species with lattice spacings corresponding to those of Li_3_NbO_4_ were detected within an amorphous surface layer using nanobeam electron diffraction (NBED) [18]. However, such small crystalline particles cannot be identified using X‐ray or synchrotron diffraction.

### 3.4 Amorphous Nature of Li–Nb Coatings in Sol–Gel‐Processed NMC811 at 500°C

Notably, a similar sol–gel method was employed in a separate study to coat the same material, NMC811 (ECOPRO), with Li–Nb oxides [19]. After calcination at 500°C, XRD analysis revealed no detectable crystalline LiNbO_3_, even for samples with a 3 wt.% Li‍–‍Nb‐containing phase. If the coated concentration of Li–Nb oxide is 3% (elemental analysis is not provided), then at 500°C, crystalline LiNbO_3_ is forming, and at least two weak reflections at 2*θ* ≈ 23.8 (012) and 32.8°(104) (Cu Kα radiation) would be expected. This is not the case. One explanation could be too thin Li–Nb oxide coating, which could not be detected by in‐house XRD instruments. Second explanation is that for this “specific” coating reaction [19] (Ref. [19]: use of lithium–niobium ethoxide solution (5% w/v in ethanol) versus Ref. [10], when just Nb ethoxide in ethanol was used), the calcination temperature, 500°C, is too low and LiNbO_3_ is not yet crystalline to be observed by diffraction. The first explanation can be excluded because, in both papers, Ref. [10] and Ref. [19], the same NMC811 (ECOPRO) was used. Moreover, the coated samples were calcined at the same temperature (500°C) in pure oxygen, and measured with the same XRD instrument. In addition, in Ref. [10], the XRD patterns of the 1.4 wt.% Nb‐modified NMC811X sample show clear reflections at 2*θ* ≈ 2.5° (012) and 3.5° (104) for LiNbO_3_ (synchrotron radiation, *λ *= 0.18266 Å). For the 0.7 wt.% Nb‐modified sample (measured using an in‐house XRD instrument, D8 ADVANCE (Cu Kα radiation)), the patterns also exhibit sharp reflections corresponding to the LiNbO_3_ structure [10]. Therefore, one possible explanation is that under the specific coating conditions used in this study, namely, lithium‐niobium ethoxide solution (5% w/v in ethanol) and calcination at 500°C, the Li–Nb‐containing phase remains predominantly amorphous or nanocrystalline. Note that the formation of nanocrystalline LiNbO_3_ and Li_3_NbO_4_ is not excluded. As was recently pointed out, LiNbO_3_ and Li_3_NbO_4_ could be present in the surface layer already at 475°C, but they are difficult to detect because of their low crystallinity [11].

### 3.5 Li–Nb‐Coated NMC9055 and Ni90533: Evidence Suggests Unoptimized Surfaces and Unresolved Phase Identity

Bragg reflections from the Li–Nb oxide coating were also detected for other sol–gel‐based syntheses of noncommercial nickel‐rich NMC, NMC9055 (Ni : Mn : Co: 90% : 5% : 5%) [20]. For the synthesis of this Li–Nb‐coated NMC9055 cathode material, first Nb‐coated/doped (Ni_0.90_Mn_0.05_Co_0.05_)(OH)_2_ was obtained, then it was mixed with LiOH·H_2_O and sintered in pure oxygen atmosphere for 20 h at 725°C. Unfortunately, there are no temperature‐dependent XRD measurements between 350 and 725°C—the coated material was annealed only at 725°C. At this temperature, with increasing Nb concentration till 2.1 wt.%, some extra reflections are spotted at 2*θ* ≈ 5.1 (400) and 7.2° (440) (synchrotron radiation, *λ *= 0.16625 Å), which are assigned to Li_3_NbO_4_. Indeed, the reflections at these angles are typical for Li_3_NbO_4_, but to correctly assign them to Li_3_NbO_4_ more details are needed, for example, the presence of other reflections (between 2*θ* ≈ 2.5° and 4.0°) and a detailed temperature‐dependent XRD measurement. This is not the case. The attempt to justify/rationalize the assignment of the spotted extra reflections to Li_3_NbO_4_ by mixtures of Nb precursor and LiOH mixed with a molar ratio of 1:1, 1:3, and 1:5 and sintered at 725°C in O_2_ in the absence of the NMC9055 cathode material was a reasonable decision [20]. However, we must consider that when these mixtures are calcined, their crystallization conditions differ significantly from the crystallization that occurs on the surface of the cathode‐active materials [18]. Moreover, it has to be considered that in such coated compounds at temperatures higher than ~630°C, Li–Nb oxides may react with the active material, resulting in different compounds (for example, *x*Li_3_NbO_4_−(1−*x*)LiMO_2_). A logical question arises regarding what is occurring in the temperature range of 350–725°C. In our opinion, for this Li–Nb oxide‐coated NMC9055 compound, no detectable LiNbO_3_ reflections are observed within this temperature range. If so, the assignment of the synchrotron XRD reflections at 2*θ* ≈ 5.1 (400) and 7.2° (440) to Li_3_NbO_4_ may also warrant further consideration. Unfortunately, an enlarged view of the 2*θ* ≈ 2.5°–4.0° region, where two broad XRD peaks corresponding to Li_3_NbO_4_ would be expected, is not provided. This leads us to conclude that the compound can be regarded as a self‐synthesized and unoptimized material, exhibiting reactivity and stability similar to those of NMC622PC and NMC811SS. Specifically, the surface appears unstable, and at 725°C, the active NMC phase may react with Li/Nb compounds, potentially resulting in a doped NMC material and a cation‐disordered crystalline rock‐salt phase (*x*Li_3_NbO_4_−(1−*x*)LiMO_2_) [16]. As with other examples, the presence of nanocrystalline LiNbO_3_ and Li_3_NbO_4_ cannot be excluded.

Recently, another example of a self‐synthesized and unoptimized Nb‐modified Ni‐rich layered cathode material, Ni90533 (Ni:Mn:Co: 90%:5%:3.3%), was synthesized by heating a mixture of metal NMC hydroxide, (Ni_0.90_Mn_0.05_Co_0.033_)(OH)_2_, Nb ethoxide, and LiOH at 775°C in oxygen [21]. In the region between 1.5° and 4°, the synchrotron XRD pattern shows some weak reflections, but they could not be attributed to any known phase.

### 3.6 Phase Evolution Differences Between Optimized and Unoptimized NMC Cathodes

Based on the above‐presented examples and their comparison, it can be concluded that between ~400 and 800°C, only two extra reflections (at 2*θ* ≈ 43° and 63°, using Cu *K*_α_ radiation) are observed for our self‐synthesized and commercially unoptimized coated materials (NMC811SS and NMC622PC). In contrast, for certain commercial materials (NMC811X [10, 11] and NMC622SC) with unknown synthetic history, all types of reflections, corresponding to both LiNbO_3_ and Li_3_NbO_4_, are detected. Several factors may contribute to this difference:

1. for NMC811SS and NMC622PC materials, between ~400 and ~650°C, both phases, LiNbO_3_ and Li_3_NbO_4_, are amorphous or nanocrystalline and cannot be determined by X‐ray or synchrotron diffraction;
2. above ~650°C, only the second phase, Li_3_NbO_4_, begins to crystallize into larger crystals, and additional reflections become visible. However, it remains unclear why the reflections at 2*θ* ≈ 15°, 25°, and 33° are not detected, while only those at 2*θ* ≈ 43° and 63° are observed. One possible explanation for this effect is that, starting around 650°C, the formation of a rock‐salt‐type solid solution, *x*Li_3_NbO_4_‐(1−*x*)LiMO_2_, begins, with the value of *x* being temperature‐dependent. The variation in *x* affects the position of some reflections, causing peak shifts and overlap with those of the active material, which makes precise identification and refinement difficult. However, at 800 and 1000°C, the diffraction pattern can be well explained, including the rock‐salt phase *x*Li_3_NbO_4_−(1−*x*)LiMO_2,_ and allows a refinement of its structure (Figures S2e,f and S4a,b). To assess the validity of the phase assignments in the 400–650°C range and to accurately identify all present species, advanced characterization techniques are necessary.
3. for the commercial NMC811X [10, 11] and NMC622SC materials, the surface’s stability is most probably improved, and the Li–Nb oxide crystallization process may take place on a surface with lower chemical reactivity toward the coating precursors, favoring the crystallization of both oxides, LiNbO_3_ and Li_3_NbO_4_;
4. For the NMC811X material, the highest calcination temperature applied was 815°C [11]. The potential formation of the rock‐salt phase *x*Li_3_NbO_4_−(1−*x*)LiMO_2_ at higher temperatures was not investigated; therefore, its presence cannot be entirely ruled out. In the case of NMC622SC, the rock‐salt *x*Li_3_NbO_4_‐(1−*x*)LiMO_2_ phase is clearly observed only at 1000°C, indicating greater thermal stability of NMC622SC compared to NMC622PC. However, the presence of this phase at 800°C cannot be excluded, although it would likely be in smaller quantities.

### 3.7 Commercial NMC Materials: Optimization Does Not Guarantee Crystalline Li–Nb Phases

It is important to note that the use of commercially available materials, such as those from ECOPRO or MSE Supplies, does not necessarily indicate that they are fully optimized or that they will exhibit crystalline Li–Nb oxides in XRD patterns. For comparison, we also examined another NMC material from MSE Supplies, LiNi_0.82_Mn_0.07_Co_0.11_O_2_ (Ni82). Similar to NMC622PC and self‐synthesized NMC811SS, XRD analysis of Ni82 coated with 5 wt.% Li–Nb oxide and calcined at temperatures ranging from 350 to 800°C did not reveal the presence of any crystalline LiNbO_3_ phase (Figure S5). We suspect that this material incorporates zirconium and is coated with borate, as it is sourced from the same supplier and has a composition similar to LiNi_0.83_Mn_0.06_Co_0.11_O_2_ (Ni83) (MSE Supplies), for which an X‐ray photoelectron spectroscopy (XPS) survey scan confirmed the presence of these elements (Zr and B) [22]. However, this type of optimization did not improve the surface stability of the sample sufficiently to promote the crystallization of Li–Nb oxides within the considered temperature range.

### 3.8 High‐Temperature Route Toward Conductive Rock‐Salt Surface Phases

Finally, regarding the results we obtained from studying the coating of NMC622PC, NMC622SC, and NMC811SS at high temperatures, for NMC622PC and NMC811SS, there are still more questions than answers. However, for NMC622SC, upon increasing the temperature to 1000°C, a Li_3_NbO_4_‐based system with a cation‐disordered rock‐salt structure, *x*Li_3_NbO_4_−(1−*x*)LiMO_2_, is formed. Although this latter phase is not clearly visible in the X‐ray diffraction pattern obtained at 900°C, it is nevertheless suggested that it is most likely present alongside Li_3_NbO_4_ at this temperature. Notably, while both LiNbO_3_ and Li_3_NbO_4_ are electronic insulators, the *x*Li_3_NbO_4_−(1−*x*)LiMO_2_ solid solution may exhibit enhanced electronic conductivity due to the partial substitution of Nb^5+^ and Li^+^ with transition metal ions, which can introduce mixed‐valent states and/or partially filled d‐orbitals. Therefore, assuming that NMC622SC remains structurally stable at 900–1000°C (despite an observed Li/Ni mixing of approximately 11%), the formation of a surface layer of *x*Li_3_NbO_4_−(1−*x*)LiMO_2_ could be of interest for further investigation as a surface‐reconstructed cathode material. Although it is more difficult to crystallographically confirm the LiNbO_3_ → Li_3_NbO_4_ → *x*Li_3_NbO_4_−(1−*x*)LiMO_2_ transition in NMC622PC, the data obtained for this material in the temperature range of 800–1000°C also indicate good potential as a promising candidate. This approach may offer opportunities for the development of advanced surface‐coated electrode materials derived from cathode active materials (CAMs) specifically optimized for stability at high temperatures.

### 3.9 Identifying the Li–Nb–M–O Rock‐Salt Phase in NMC622: Dismissing NiO‐Like Domains as a Possible Source

In order to further support the conclusions drawn from the in‐house XRD measurements, the structural characteristics of 6 wt.% Li–Nb oxide‐coated NMC622SC and NMC622PC samples were additionally investigated by synchrotron X‐ray diffraction (Figure S3). The obtained diffraction patterns are in good agreement with those measured using laboratory instrumentation, confirming the validity of the conclusions derived from the in‐house XRD data. In particular, the sharp reflections observed for the NMC622PC sample calcined at 800°C (Figure S3b), which were assigned to a rock‐salt‐type *x*Li_3_NbO_4_−(1−*x*)LiMO_2_ phase, provide strong evidence for the formation of this phase rather than an alternative decomposition product.

It should be noted that these reflections can, in principle, also be indexed to a rock‐salt‐type NiO phase (Fm‐3m). The gradual increase in their intensity with increasing calcination temperature (e.g., at 800 and 1000°C, Figures 1b and S3b) could therefore be interpreted as the formation of NiO‐like domains, possibly originating from surface transformation of the layered NMC structure. Furthermore, at high calcination temperatures, the formation of a mixture of transition‐metal monoxides (NiO, CoO, and MnO) cannot be excluded a priori.

However, it is important to consider that the investigated systems are not binary but multicomponent in nature, comprising Li, Nb, Ni, Co, Mn, and O ions. In such systems, the formation of separate NiO, CoO, and MnO phases or a random (Ni,Co,Mn)O solid solution would be expected to result in pronounced peak broadening or peak splitting due to the substantially different lattice parameters of the individual monoxides (e.g., NiO: *a* ≈ 4.17 Å, CoO: *a* ≈ 4.24 Å, MnO: *a* ≈ 4.44 Å). Consequently, once Li^+^ and Nb^5+^ ions participate in the reconstructed phase, a simple MO‐type oxide is no longer a realistic final state.

In contrast, the experimentally observed reflections (Figures 1b and S3b) remain relatively sharp and exhibit only minor peak shifts, which is inconsistent with a disordered mixture of transition‐metal monoxides. This behavior is instead consistent with the formation of an Nb‐stabilized Li–Nb–M–O rock‐salt‐type surface phase, such as *x*Li_3_NbO_4_−(1−*x*)LiMO_2_ (M = Ni, Co, Mn), in which Nb incorporation can stabilize the rock‐salt‐type framework over a range of compositions. As reported previously, the characteristic reflections of these rock‐salt phases appear at approximately 37°, 43°, 63°, 75°, and 79° (Cu *K*_α_ radiation), corresponding to the (111), (200), (202), (311), and (222) planes, respectively. Because *x*Li_3_NbO_4_−(1−*x*)LiMO_2_ represents a solid‐solution series, the exact lattice parameter and peak positions depend on both the Nb content (*x*) and the transition‐metal composition, resulting in a limited range of diffraction positions rather than a single fixed value. Similar limited peak shifts and sharp reflections have been reported previously for Li–Nb‐transition‐metal oxide solid solutions [16]. Therefore, the observed reflections are attributed to a Nb‐influenced Li–Nb–M–O surface phase rather than to simple transition‐metal monoxides.

### 3.10 Critical Assessment of Reported Li–Nb Oxide Phases in Sol–Gel‐Derived Coatings on NMC811 Materials

During our journey in search of answers to our questions, we came across some findings in the literature that were not entirely clear. In the following discussion, we analyze several publications in which the reported XRD measurement results give rise to noteworthy questions.

Reflections from Li–Nb oxide coating are also noticeable in several other sol–gel‐based approaches [14, 15, 23]. Typically, to obtain sol–gel‐derived coatings, cathode active materials are suspended in alcohols together with Nb‐ and Li‐alkoxides. Such a process is also possible to make in water using a water‐soluble niobium ammonium oxalate (NH_4_)_3_[NbO(C_2_O_4_)_3_] (NAmO_*x*_) [14]. An NMC811 was synthesized from commercial Ni_0.8_Co_0.1_Mn_0.1_(OH)_2_ precursor and LiOH⋅H_2_O. The obtained material was then coated with Li–Nb oxide using NAmOx dissolved in deionized water. The resulting powder, NMC811N, was then sintered at 550°C. Since the final product was obtained from a self‐synthesized and unoptimized NMC material, its XRD patterns are not expected to show any LiNbO_3_ diffraction peaks. This is indeed the case for the 0.5–2 wt.% LiNbO/NMC811N samples. However, when the amount of coated Li–Nb oxide was increased to 5%, several additional diffraction peaks appeared at 2*θ* ≈ 23.7° (012), 32.7° (104), and 42.8° (202) (Cu *K*_α_ radiation), which were attributed to LiNbO_3_.

Several features are questionable in case of this sample. Based on the literature survey and our results, the reflection observed at 2*θ* ≈ 42.8° appears too strong to be attributed to LiNbO_3_ coated on NMC811 and calcined at 550°C. Usually, such a diffraction peak, in combination with one located at 2*θ* ≈ 62.5° (440) (clearly seen in Figure S1 of the examined paper [14]), is attributed to Li_3_NbO_4_ observed at temperatures higher than ~650°C. 550°C is below the onset of crystallization for Li_3_NbO_4_; at this temperature, Li_3_NbO_4_ is not yet crystalline to be detected by an in‐house diffractometer or synchrotron diffraction. Another doubt is related to the reflections at 2*θ* ≈ 23.7° and 32.7°, which are also assigned to LiNbO_3_. They are too narrow to be ascribed to LiNbO_3_ in combination with the reflection at 2*θ* ≈ 42.8°. Moreover, if these two reflections are detected, then why are others, for example, at 2*θ* ≈ 34.5° (104) and/or 53° (116), not observed? A Rietveld refinement would have clarified these two issues. Taking into consideration the somewhat ’special’ coating method presented in this paper, a most plausible explanation is that the reflections detected for NMC811N coated with 5 wt.% Li–Nb oxide correspond to an unknown phase or phases.

### 3.11 Reevaluation of Li–Nb Oxide Phase Formation on Commercial NMC622 Coated Using Ammonium Niobium Oxalate

A similar ambiguity arises in other studies employing ammonium niobium oxalate as the niobium source for coating commercial NMC622, where the reported XRD reflections again suggest inconsistencies in the assigned Li–Nb oxide phases. For example, in a recent study, a new attempt was made to avoid moisture‐sensitive and expensive niobium ethoxide in the coating process of another nickel‐rich cathode active material, NMC622. As niobium source, ammonium niobium oxalate NH_4_[NbO(C_2_O_4_)_2_(H_2_O)_2_]⋅3H_2_O, (ANO) was used as active material—commercial LiNi_0.6_Co_0.2_Mn_0.2_O_2_ powder (NCM622, Wujie Technology) [15]. The resulting coated powder, NMC622WT, was then sintered at 550°C. As in case of previously discussed material (NMC811N) [14], several coated samples with different Li–Nb oxide concentrations were prepared: 0.5–2 wt.% LiNbO/NMC622WT. Additionally, a 20 wt.% Li–Nb oxide‐loaded sample (stated to be as reference for structural analysis) (20 wt.% LiNbO/NMC622WT) was prepared with the aim of reaching sufficient coating to observe XRD reflections from Nb‐containing phases. Although no detailed information about the synthetic history and postsynthetic treatment of the commercial NCM622 used in this study is provided, analyzing the published results by other groups and our unpublished ones, we conclude that this material should show similar crystallographic behavior to NMC622PC (BASF) (Figure 1b) studied by us. In our opinion, this material (20 wt.% LiNbO/NMC622WT) after temperature treatment at 550°C should not show any additional diffraction peaks, except those for the active material, NMC622. For the 20 wt.% LiNbO/NMC622WT sample, however, additional reflections at 2*θ* ≈ 23.7°, 42.8°, and 62.5° (Cu *K*_α_ radiation) were observed. The reflections at 2*θ* ≈ 23.7° (112) and 62.5° (018) were assigned to LiNbO_3_, while the one at ~42.8° was assigned to niobium(V) oxide (Nb_2_O_5_). In our opinion, the diffraction peak at 23.7° is correctly assigned; however, it originates not from the coating itself but rather from agglomerated species (LiNbO_3_) that crystallize on the very thick, amorphous, and poorly crystalline Li–Nb oxide coating. If the main coating had been crystalline, the intensity of the reflection at 2*θ* ≈ 23.7° would have been very strong (much stronger than the observed one). The reflections at 2*θ* ≈ 42.8° and 62.5° most likely belong to Li_3_NbO_4_ or rock‐salt, *x*Li_3_NbO_4_−(1−*x*)LiMO_2_. However, this assignment is also questionable because these two oxides are not expected to form sufficiently crystalline at 550°C to be detected by X‐ray diffraction. The assignment of the diffraction peak at 2*θ* ≈ 42.8° to Nb_2_O_5_ also provokes many questions. A simple Rietveld refinement based on the diffractogram for the 20 wt.% LiNbO/NMC622WT sample could have clarified the situation.

### 3.12 Discrepancies in Reported LiNbO_3_ Reflections for Heavily Coated NMC523

An additional attempt to find answers to our questions was made by comparing our results not only with those obtained for Ni‐rich NMC compounds but also with those for “non‐Ni‍‐‍rich” NMC compounds. Again, using an aqueous sol–gel‐based approach, a commercial NMC, NMC523 (LiNi_0.5_Co_0.2_Mn_0.3_O_2_) (BASF SE), was coated with Li–Nb oxide using ammonium niobate oxalate dissolved in a mixture of glycol, ammonium citrate, and deionized water. The resulting coated powder, NMC523N, was then sintered at 900°C to obtain Li–Nb oxide‐coated cathodes with different Li–Nb oxide concentrations, 0.5, 1, and 5 wt.% [23]. Based on our experience with commercial materials from BASF SE, we assume that the CAM used in this study was also not optimized. Therefore, even for the 5 wt.% LiNbO/NMC523N‐coated sample calcined at 900°C, no reflections for LiNbO_3_ were expected in XRD patterns. This conclusion is not only based on our experience with nonoptimized NMC materials but also on the belief that the calcination temperature is too high to observe LiNbO_3_. Even though LiNbO_3_ can exist at lower temperatures (between 450 and approximately 800°C, as shown in Figure 1a, NMC622SC), at 900°C and higher, it will react with the active cathode material, resulting in Nb‐doped NMC523 and crystalline Li_3_NbO_4_ or *x*Li_3_NbO_4_−(1−*x*)LiMO_2_. Surprisingly, in the main paper discussing the XRD pattern of 5 wt.% LiNbO/NMC523N, the reflections are clearly identified at 2*θ* ≈ 23.7° (012) and 32.7° (104) (Cu *K*_α_ radiation), indicating the presence of crystalline LiNbO_3_ in the studied material. However, the Rietveld refinement results based on the XRD pattern for the same sample, presented in the Supporting Information [23], do not align with the results discussed in the main paper. This discrepancy raises questions about the validity of the XRD studies conducted on samples with excessive coating.

In conclusion, efforts to resolve the questions arising from our XRD investigations, particularly through comparison with existing literature, were largely unsuccessful or only partially effective. Therefore, a more comprehensive review of the literature was conducted.

### 3.13 Influence of Coating Method on LiNbO_3_ Crystallization and Phase Identificaton: Sol–Gel Versus Dry Coating Approaches

As an alternative method for applying a conformal LiNbO_3_ coating on CAM materials, a costly atomic layer deposition (ALD) technique could be utilized. However, this method is very expensive, and the researchers are looking for alternatives. A cheap alternative to the sol–gel method is known as dry coating, in which preformed crystalline niobium oxide, Nb_2_O_5_, is mixed with cathode‐active materials or their precursors, and the resulting mixtures are subjected to high‐temperature sintering. Recently, this method was used to coat NMC and other isostructural materials, including LiNi_0.83_Mn_0.06_Co_0.11_O_2_ (NMC831) [12] and LiNi_0.8_Mn_0.2_O_2_ (NM82) [13], with LiNbO_3_ oxide. Interestingly, although the active materials were self‐synthesized and not optimized, the characteristic reflections for crystalline LiNbO_3_ at low angles were easily observed after coating and calcination at 500°C. Also, an increase in niobium content in the Li–Nb oxide coating resulted in a shift of the CAM reflections to lower angles, indicating increased doping of Nb^5+^ into the crystalline structure [13]. It seems that, in a dry coating process, the chemistry between the CAM and Li–Nb oxide components, as well as the crystallization processes of the coating, closely resemble those that occur during the sol–gel processes. However, when a self‐synthesized and unoptimized CAM material in a sol–gel reaction is used, the presence of a solvent (water or alcohol) appears to influence the crystallinity and dimensionality/size of the coating particles. This complicates the identification of species crystallizing on the surface of the CAMs (e.g., NMC622PC and NMC811SS). So, we can conclude that in order to obtain complete information about the structure of the coating materials using XRD, it is important not only to consider the state of the active material but also the method used for coating. This does not mean that dry coating is the best choice. Controlling the morphology and uniformity of the coating is often known to be challenging in solid‐state reactions. The sol–gel method, which typically employs chemically homogeneous wet mixtures of precursors, offers certain advantages for producing coated materials; however, even this method does not guarantee a highly uniform and stable coating.

### 3.14 Contrasting Li–Nb‐Containing Phase Outcomes on LiCoO_2_ and Nickel‐Rich Layered Oxides

To complement our observations on NMC materials, we also examined published results on LiCoO_2_ coated with Li–Nb oxides, as this layered oxide offers a useful reference point for understanding coating behavior. It is well known that the layered oxide LiCoO_2_ has played a significant role in the development of lithium‐ion battery technology [24]. Due to the issues associated with cobalt, researchers are exploring alternative materials for battery cathodes, such as nickel manganese cobalt (NMC) oxides. However, in our quest to find answers to our questions, we decided to analyze the published results regarding the chemistry of this layered oxide and its coating with Li–Nb oxides as well. Recently, Zhang et al. reported on both commercial and self‐synthesized LiCoO_2_ coated with Li–Nb oxides. Surprisingly, in both cases, well‐defined crystalline LiNbO_3_ and Li_3_NbO_4_ were identified at temperatures between 400 and 800°C [25, 26]. In the first case, coated LiCoO_2_ material was synthesized using the sol–gel method, with commercial LiCoO_2_ (Hunan) as the pristine material and niobium ethoxide and Li_2_CO_3_ as the starting materials for the coating layer. In the second case, LiCoO_2_ was self‐synthesized via a high‐temperature solid‐state method using Li_2_CO_3_ and Co_3_O_4_ and then coated with Li–Nb oxides using the same sol‍–‍gel method. For both coated compounds, a crystalline LiNbO_3_ coating layer is formed in the temperature range of 400–600°C. At temperatures between 600 and 700°C, the formed LiNbO_3_ begins to react with LiCoO_2_. As the annealing temperature rises to 700 and 800°C, the degree of reaction between LiNbO_3_ and LiCoO_2_ becomes more complete, resulting in the formation of a Li_3_NbO_4_‐coating layer. Unfortunately, for these two samples, no calcination at 1000°C was performed. However, similar to the cases of NMC622SC and NMC622PC, we assume that the formation of rock‐salt *x*Li_3_NbO_4_−(1−*x*)LiCoO_2_ occurs at this temperature. This raises the question: Why does the coating process on LiCoO_2_, whether self‐synthesized or commercial, result in the formation of crystalline LiNbO_3_ and Li_3_NbO_4_, detectable by X‐ray diffraction, whereas such phases are not always, or are only selectively, observed for nickel‐rich N*_x_* M*_y_* C*_z_* materials? This difference can be attributed to several factors, primarily related to the structural, electronic, and thermodynamic properties of these materials. The different behavior of LiCoO_2_ and Ni‐rich NMC can be related to differences in their electronic structure, cation–oxygen interactions, and structural stability during delithiation and thermal treatment. In contrast, nickel‐rich NMC materials are prone to structural instability, particularly at high nickel contents, often resulting in phase transitions. In LiCoO_2_, cobalt primarily cycles between Co^3+^ and Co^4+^, whereas nickel‐based layered oxides can accommodate Ni^2+^, Ni^3+^, and Ni^4+^, reflecting the broader range of accessible oxidation states of nickel. These variations can trigger structural rearrangements and contribute to the overall instability of nickel‐rich NMC materials. Moreover, the presence of nickel tends to promote greater cation mixing and structural disorder, further compromising the material’s integrity. Additionally, LiCoO_2_ generally demonstrates superior thermal stability compared to that of its nickel‐rich counterparts. Due to these factors, additional optimization steps for stabilizing the LiCoO_2_ surface may not be necessary when the material is used in batteries or for further research.

The present results also have implications for how coating studies are interpreted. It is common to conclude that a particular coating strategy is effective for “Ni‐rich layered oxides” or “layered oxide cathodes.” However, the present results and literature analysis suggest that such general conclusions should be drawn with caution. A coating method demonstrated on one commercial or laboratory‐synthesized cathode cannot automatically be assumed to behave identically on another material with the same nominal composition. Instead, the conclusions should be limited to the specific cathode material investigated unless the coating strategy has been validated on multiple well‐characterized materials of different origins.

## 4 Conclusions

Based on the reported literature and our comparative analysis, several key conclusions can be drawn:

a. Whether a crystalline LiNbO_3_ or Li_3_NbO_4_ phase is formed depends on the specific NCM composition used and, more broadly, on the type of layered oxide material.
b. These phases are predominantly observed for optimized commercial materials (though not always), when a dry coating is applied, as well as in LiCoO_2_. The reasons for this are the prior surface treatment, the coating methodology, and the structural‐electronic characteristics of the coated materials.
c. Self‐synthesized materials investigated in this work react with LiNbO_3_ phase and, at higher temperatures, form the rock‐salt phase, which can also occur in commercially optimized materials at temperatures above 800°C.
d. For the successful development of coated materials, understanding the synthesis history and processing background of the NCM material is essential.

It should be noted that the structural conclusions presented here are based on samples coated with 5–6 wt.% Li–Nb oxide. This increased coating amount was intentionally selected to enable reliable detection and identification of Li–Nb‐containing crystalline reaction products by laboratory X‐ray diffraction. Although this quantity is much higher than the coating loadings typically employed for electrochemical applications (≈0.5 wt.%), it does not affect the central conclusion of this work, namely, that the crystallization pathway strongly depends on the nature and previous history of the cathode material.

While these conclusions clarify how coating behavior depends on the material’s origin and treatment, they still do not fully resolve our central question: why does coated NMC622SC show LiNbO_3_ and Li_3_NbO_4_ phases in XRD, whereas coated NMC622PC and NMC811SS do not? The purpose of literature is to inform and guide us in improving our understanding or finding answers to our questions. However, as we have seen, it is not always possible to find clear answers, as some published results are questionable. Therefore, through this article, we aimed to convey a message to researchers working in this field: do not rely on materials you do not have full information about their synthesis and treatments. The most reliable approach is to synthesize your own active materials‍—‍only then will you truly understand the processes occurring during coating and the reasons behind their good or poor electrochemical performance.

At first glance, this may appear to be a minor question regarding the crystallinity of the coating. However, addressing such questions is essential for understanding the enhanced stability of the cathode‐active material in batteries, as well as the underlying chemistry and interactions between the cathode material and the coating or electrolyte. Insights gained from such investigation may also help answer broader questions, such as whether this interaction is direct or mediated by an additional coating applied during industrial production. The enhanced stability of commercial materials may result from proprietary optimization processes that are not disclosed in the public literature. If researchers intend to use these materials for further investigation, they must contact the producer to request comprehensive details. However, this may pose challenges, as the producer may be reluctant to provide such information.

Moreover, working with commercial materials can yield promising and interesting results. That is, the data may indicate that the coating performs well for a given material, and the electrochemical performance appears favorable. However, such findings do not necessarily allow us to conclude that a specific coating is universally effective for a particular active material. As our observations suggest, it is crucial to know the exact synthetic history of the material—whether self‐synthesized or commercial—in order to accurately assess its intrinsic activity. This understanding is essential for interpreting the true structure of the coating and its role in surface protection during electrochemical processes as well as to better interpret crystallographic or other experimental results that may sometimes appear unexpected or difficult to explain.

## Conflicts of Interest

The authors declare no conflicts of interest.

## Supporting information

Supplementary Material

## Data Availability

The data that support the findings of this study are available from the corresponding author upon reasonable request.
