# Supplementary material for: Influence of Cathode Origin on the Crystallization of LiNbO3 Coatings on Ni‐Rich Layered Oxides
Source: Chempluschem. 2026 Sep 16;91(9):e70248. doi: 10.1002/cplu.70248 (PMC13581342; doi:10.1002/cplu.70248)
Supplement: Supplementary file 1 [file CPLU-91-e70248-s001.pdf]

Supporting Information

for

Influence of Cathode Origin on the Crystallization of $LiNbO_3$ Coatings on Ni-Rich Layered Oxides

Valeriu Mereacre, Yiran Guo, Ruochen Xu, Anna-Lena Hansen, Michael Knapp, Helmut Ehrenberg and Joachim R. Binder

Institute for Applied Materials – Energy Storage Systems, Karlsruhe Institute of Technology, 76344 Eggenstein-Leopoldshafen, Germany

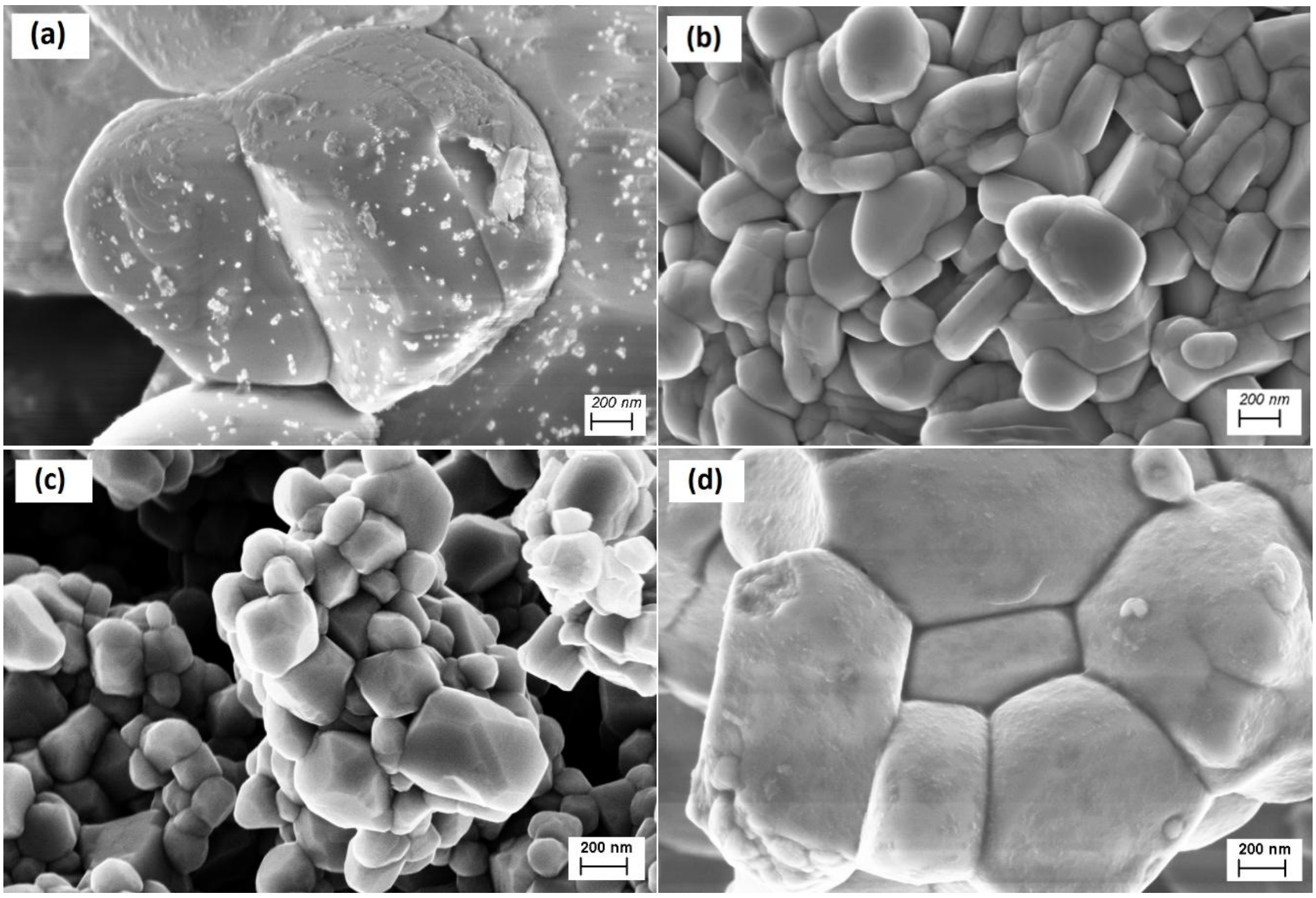

Figure S1. SEM images of the pristine, uncoated, NMC622SC (a), NMC622PC (b), NMC811SS (c) and Ni82 (d).

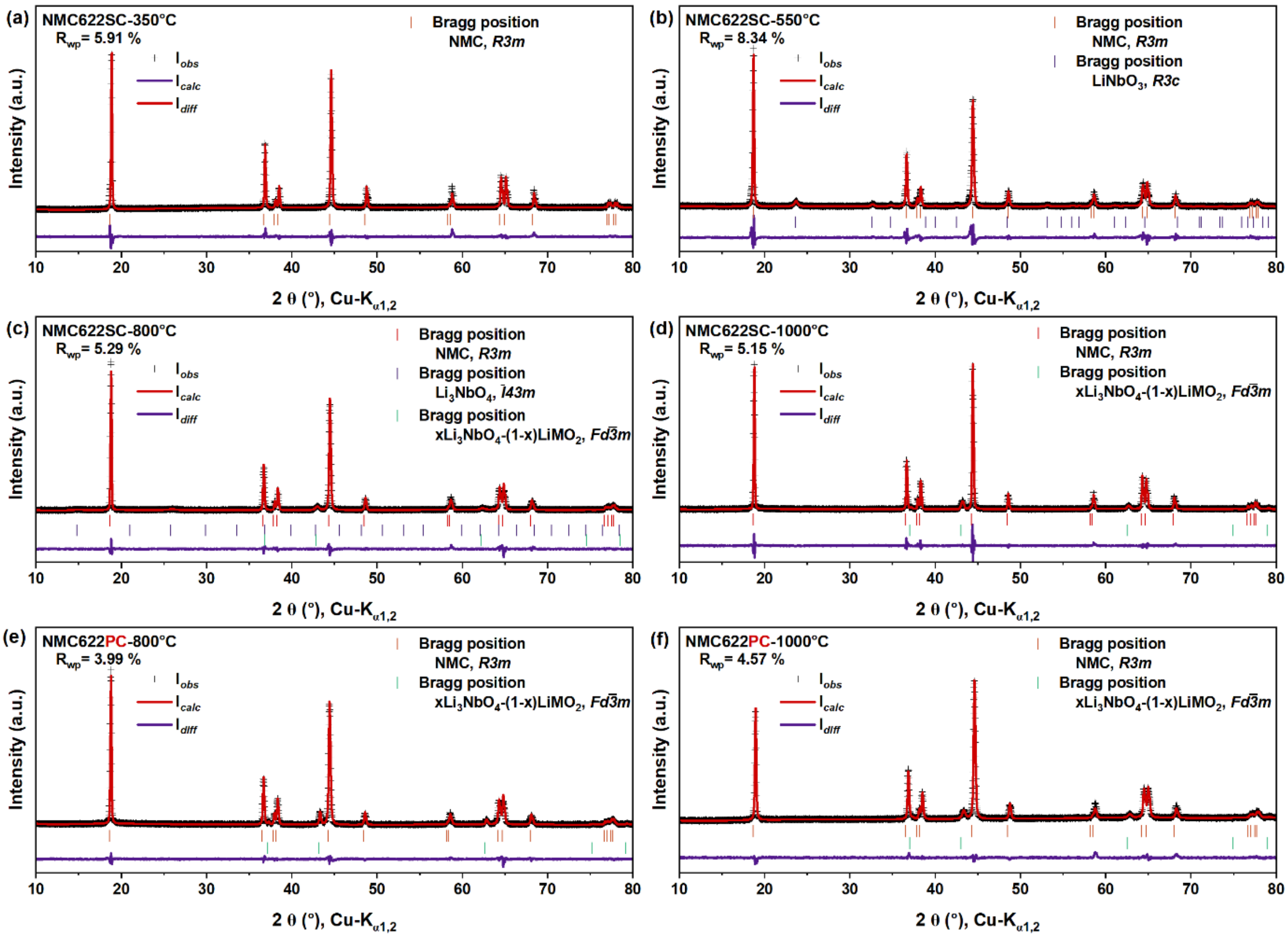

Figure S2. Observed and calculated X-ray diffraction patterns after Rietveld refinements of the 6 wt.% Li-Nb oxide coated samples NMC622SC (a-d) and NMC622PC (e,f) heated at different temperatures: 350, 550, 800, and 1000 °C.

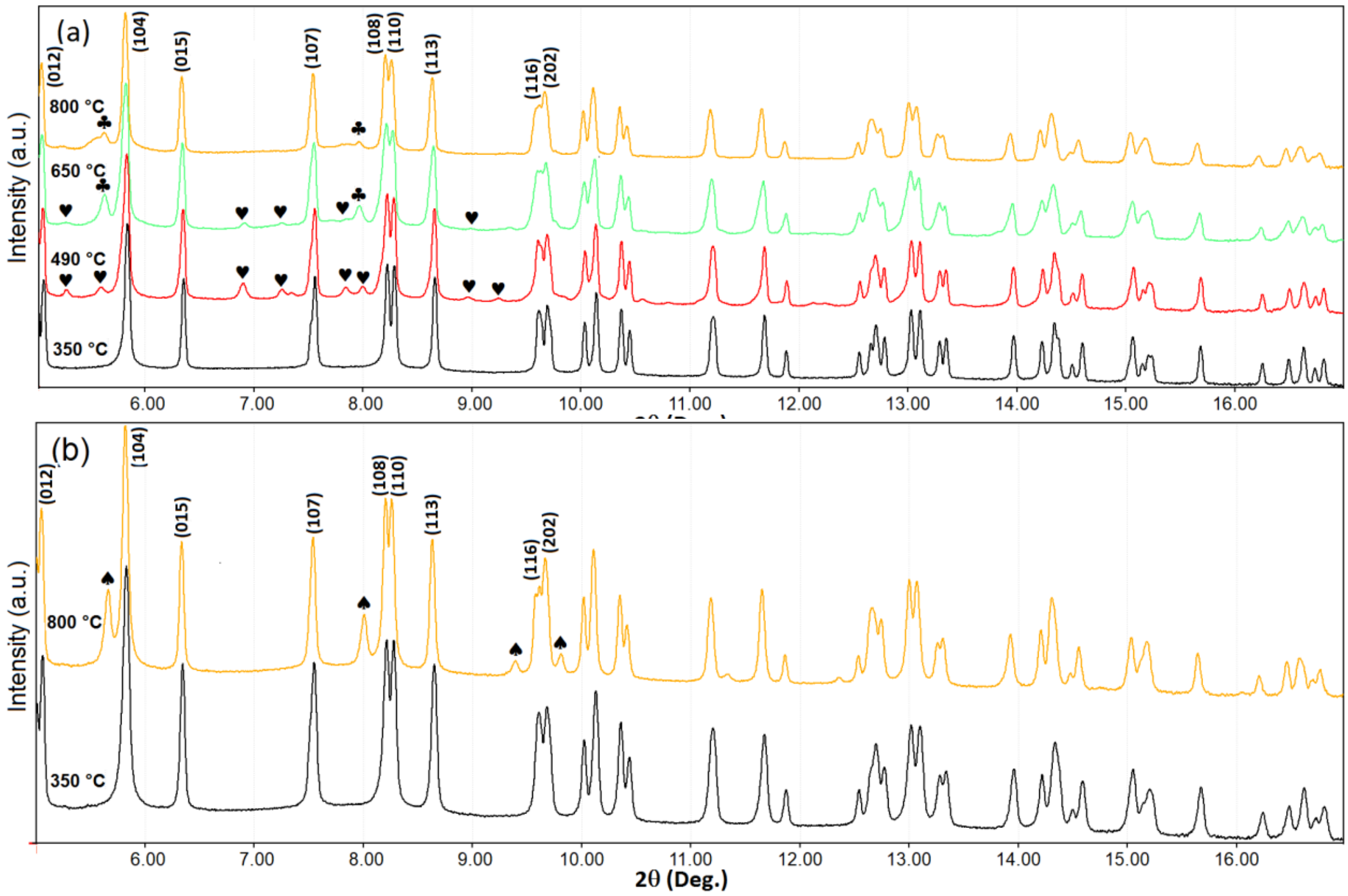

Figure S3. Synchrotron XRD patterns of 6 wt.% Li-Nb oxide coated samples NMC622SC (a) and NMC622PC (b) heated at different temperatures; ♥ is $LiNbO_3$, ♣ is Li_3NbO_4, ♠ is xLi_3NbO_4-$(1-x)LiMO_2$.

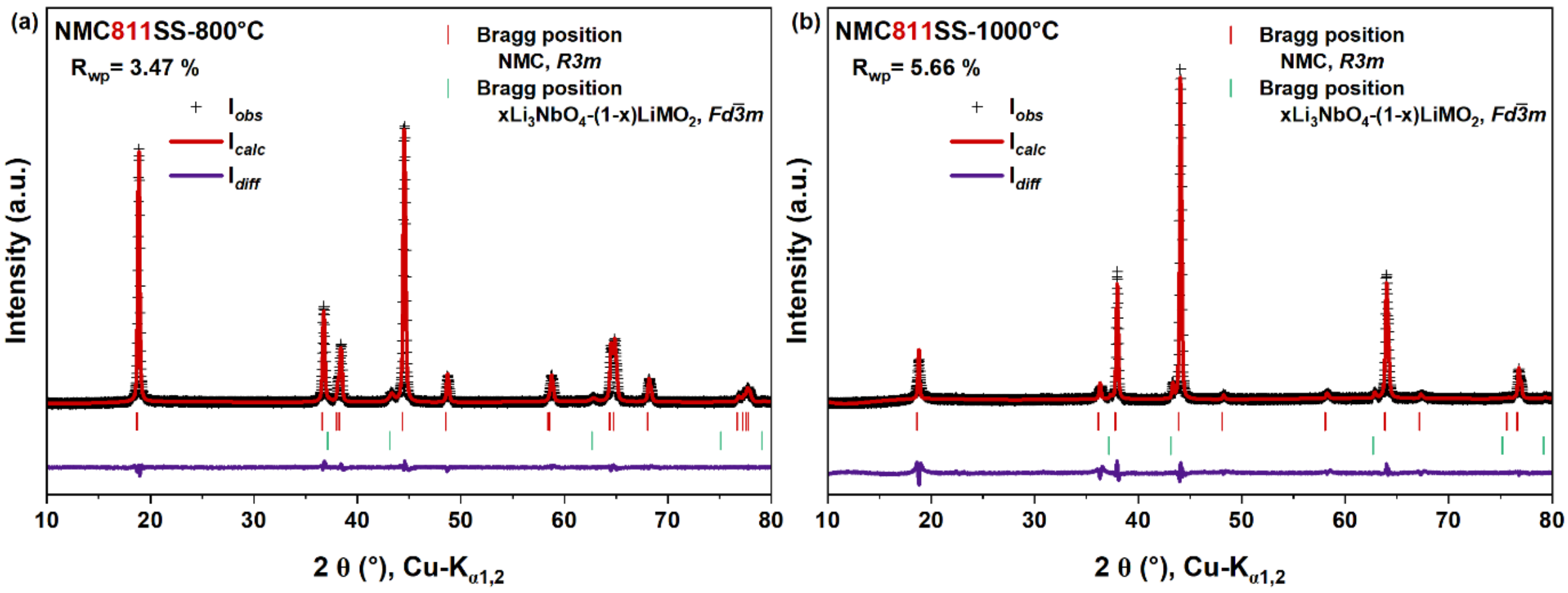

Figure S4. Observed and calculated X-ray diffraction patterns after Rietveld refinements of the 5 wt.% Li-Nb oxide coated samples NMC811SS heated at 800 and 1000 °C.

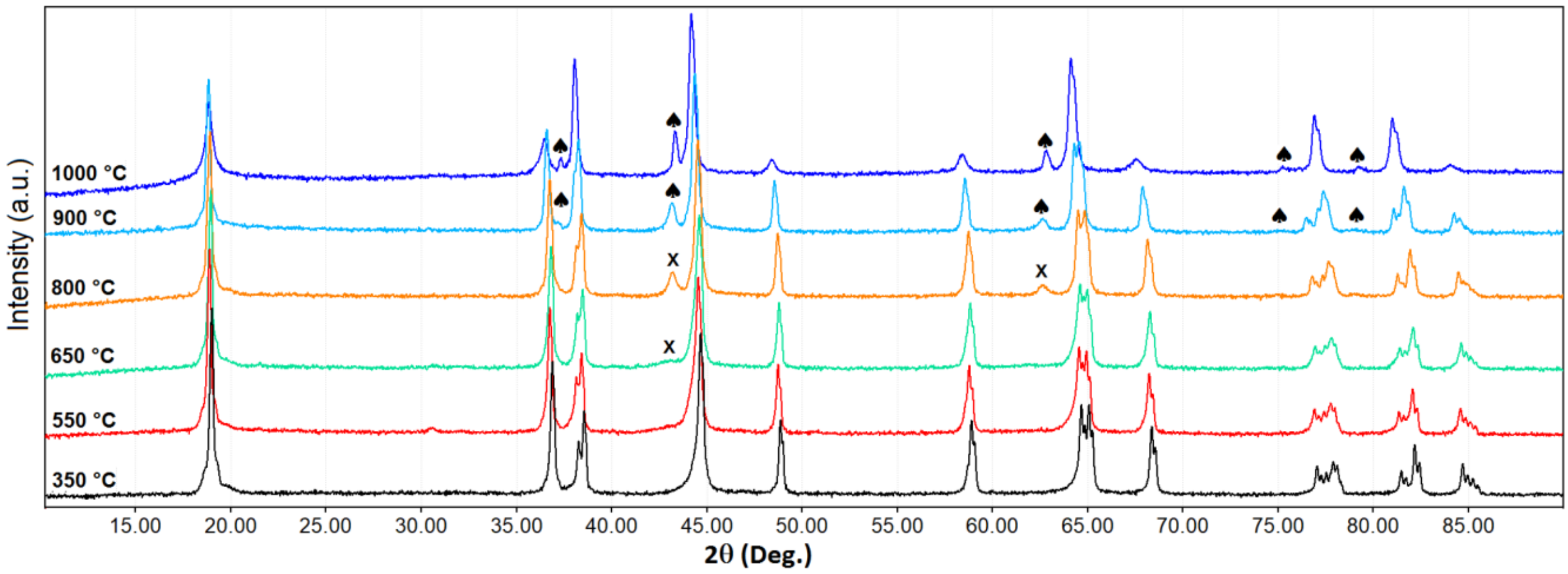

Figure S5. XRD for 5 wt.% Li-Nb oxide coated sample $LiNi_{0.82}Mn_{0.07}Co_{0.11}O_2$ (Ni82) heated at different temperatures: 350, 550, 650, 800, 900, and 1000 °C; ♠ is xLi_3NbO_4-(1-x)$LiMO_2$. The presence of Li_3NbO_4 is not excluded and **x** is most probably Li_3NbO_4 or a mixture Li_3NbO_4/xLi_3NbO_4-(1-x)$LiMO_2$.

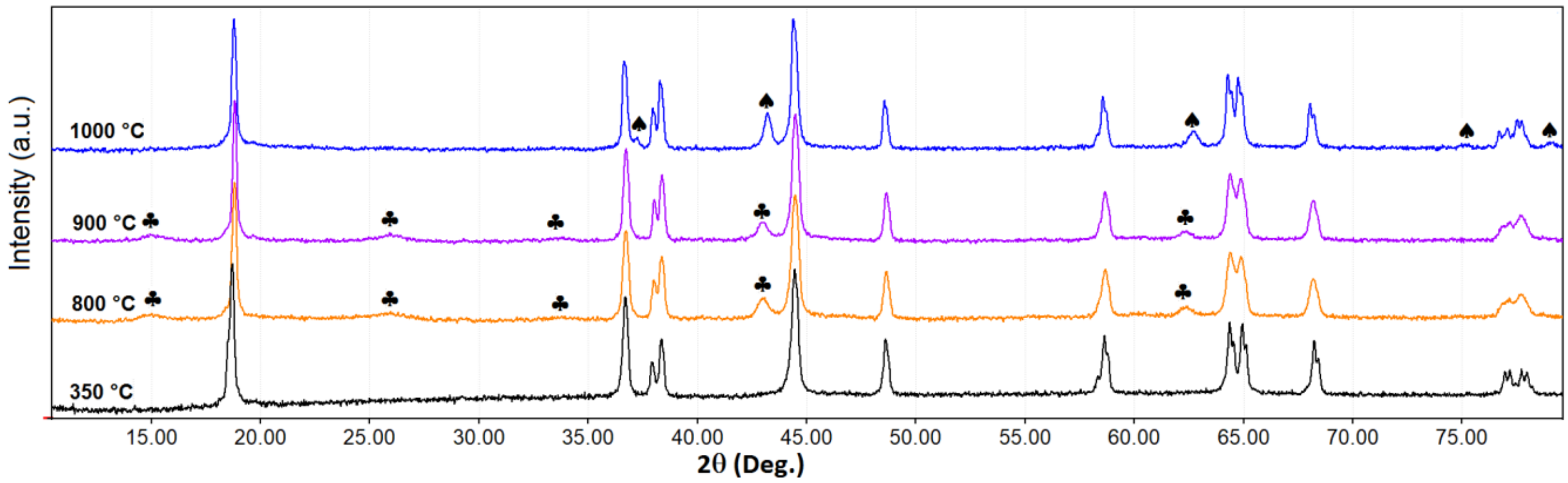

Figure S6. XRD for 6 wt.% Li-Nb oxide coated sample NMC622SC heated at different temperatures: 350, 800, 900, and 1000 °C; ♠ is xLi_3NbO_4-(1-x)$LiMO_2$, ♣ is Li_3NbO_4.

Table S1. Crystallographic parameters of 6 wt.% Li-Nb oxide coated sample NMC622SC heated at 350 °C.

Cell parameters
Space group: *R-3m*, $a = b = 2.8687$ Å, $c = 14.2238$ Å, $\alpha = \beta = 90°$, $\gamma = 120°$
Ni on Li site: 0.0663 ± 0.0034, $R_{wp} = 5.91$ %
Atomic positions

Name	site	*x*	y	z	occupancy
Li_1	3*a*	0.000	0.000	0.000	0.934
Ni_2	3*a*	0.000	0.000	0.000	0.066
Ni_1	3*b*	0.000	0.000	0.500	0.534
Li_2	3*b*	0.000	0.000	0.500	0.066
Co_1	3*b*	0.000	0.000	0.500	0.200
Mn_1	3*b*	0.000	0.000	0.500	0.200
O_1	6c	0.000	0.000	0.247	1.000

Table S2. Crystallographic parameters of 6 wt.% Li-Nb oxide coated sample NMC622SC heated at 550 °C.

Cell parameters (layered phase, 96 wt.%)
Space group: *R-3m*, $a = b = 2.8725$, $c = 14.2245$ Å, $\alpha = \beta = 90°$, $\gamma = 120°$
Ni on Li site: 0.0578 ± 0.0044, $R_{wp} = 8.34$ %
Atomic positions

Name	site	*x*	y	z	occupancy
Li_1	3*a*	0.000	0.000	0.000	0.942
Ni_2	3*a*	0.000	0.000	0.000	0.058
Ni_1	3*b*	0.000	0.000	0.500	0.542
Li_2	3*b*	0.000	0.000	0.500	0.058
Co_1	3*b*	0.000	0.000	0.500	0.200
Mn_1	3*b*	0.000	0.000	0.500	0.200
O_1	6c	0.000	0.000	0.244	1.000

Cell parameters (niobate phase-$LiNbO_3$, 5 wt.%)
Space group: *R-3c*, $a = b = 5.1533$ Å, $c = 13.8819$ Å, $\alpha = \beta = 90°$, $\gamma = 120°$
Atomic positions

Name	site	*x*	y	z	occupancy
Li_1	4*a*	0.000	0.000	0.283	1.000
Nb_1	4*a*	0.000	0.000	0.000	1.000
O_1	4b	0.049	0.344	0.065	1.000

Table S3. Crystallographic parameters of Crystallographic parameters of 6 wt.% Li-Nb oxide coated samples NMC622SC heated at 800 °C.

Name	site	*x*	y	z	occupancy
Cell parameters (layered phase, 92 wt.%)<br>Space group: *R-3m*, $a = b = 2.8778$, $c = 14.2403$ Å, $\alpha = \beta = 90°$, $\gamma = 120°$<br>Ni on Li site: 0.1033 ± 0.0024, $R_{wp} = 5.29$ %<br>Atomic positions					
Li_1	3*a*	0.000	0.000	0.000	0.897
Ni_2	3*a*	0.000	0.000	0.000	0.103
Ni_1	3*b*	0.000	0.000	0.500	0.497
Li_2	3*b*	0.000	0.000	0.500	0.103
Co_1	3*b*	0.000	0.000	0.500	0.200
Mn_1	3*b*	0.000	0.000	0.500	0.200
O_1	6c	0.000	0.000	0.244	1.000
Cell parameters (rock-salt phase, 5 wt.%)<br>Space group: *Fm-3m*, $a = b = c = 4.2177$ Å, $\alpha = \beta = \gamma = 90°$<br>Atomic positions					
Name	site	*x*	y	z	occupancy
Li_1	4*a*	0.000	0.000	0.000	1.000
Ni_1	4*a*	0.000	0.000	0.000	1.000
Nb_1	4*a*	0.000	0.000	0.000	1.000
O_1	4b	0.500	0.500	0.500	1.000
Cell parameters (niobate phase- Li_3NbO_4, 3 wt.%)<br>Space group: *I-4 3m*, $a = b = c = 8.4416$ Å, $\alpha = \beta = \gamma = 90°$<br>Atomic positions					
Name	site	*x*	y	z	occupancy
Li_1	4*a*	0.378	0.378	0.104	1.000
Nb_1	4*a*	0.140	0.140	0.140	1.000
O_1	4b	0.891	0.891	0.891	1.000
O_2	4b	0.125	0.125	0.360	1.000

Table S4. Crystallographic parameters of 6 wt.% Li-Nb oxide coated sample NMC622SC heated at 1000 °C.

Cell parameters (layered phase, 91 wt.%)<br>Space group: *R-3m*, $a = b = 2.8778$, $c = 14.2403$ Å, $\alpha = \beta = 90°$, $\gamma = 120°$<br>Ni on Li site: 0.1033 ± 0.0024, $R_{wp} = 5.14$ %<br>Atomic positions					
Name	site	*x*	y	z	occupancy
Li_1	3*a*	0.000	0.000	0.000	0.882
Ni_2	3*a*	0.000	0.000	0.000	0.118
Ni_1	3*b*	0.000	0.000	0.500	0.482
Li_2	3*b*	0.000	0.000	0.500	0.118
Co_1	3*b*	0.000	0.000	0.500	0.200
Mn_1	3*b*	0.000	0.000	0.500	0.200
O_1	6c	0.000	0.000	0.244	1.000
Cell parameters (rock-salt phase, 9 wt.%)<br>Space group: *Fm-3m*, $a = b = c = 4.1975$ Å, $\alpha = \beta = \gamma = 90°$<br>Atomic positions					
Name	site	*x*	y	z	occupancy
Li_1	4*a*	0.000	0.000	0.000	1.000
Ni_1	4*a*	0.000	0.000	0.000	1.000
Nb_1	4*a*	0.000	0.000	0.000	1.000
O_1	4b	0.500	0.500	0.500	1.000

Table S5. Crystallographic parameters of 6 wt.% Li-Nb oxide coated sample NMC622PC heated at 800 °C.

Cell parameters (layered phase, 90 wt.%)<br>Space group: *R-3m*, *a* = *b* = 2.8772, *c* = 14.2433 Å, *α* = *β* = 90°, *γ* = 120°<br>Ni on Li site: 0.1311 ± 0.0021, R_{wp} = 4.57 %<br>Atomic positions					
Name	site	*x*	y	z	occupancy
Li_1	3*a*	0.000	0.000	0.000	0.869
Ni_2	3*a*	0.000	0.000	0.000	0.131
Ni_1	3*b*	0.000	0.000	0.500	0.469
Li_2	3*b*	0.000	0.000	0.500	0.131
Co_1	3*b*	0.000	0.000	0.500	0.200
Mn_1	3*b*	0.000	0.000	0.500	0.200
O_1	6c	0.000	0.000	0.248	1.000

Cell parameters (rock-salt phase, 10 wt.%)<br>Space group: *Fm-3m*, *a* = *b* = *c* = 4.1971 Å, *α* = *β* = *γ* = 90°<br>Atomic positions					
Name	site	*x*	y	z	occupancy
Li_1	4*a*	0.000	0.000	0.000	1.000
Ni_1	4*a*	0.000	0.000	0.000	1.000
Nb_1	4*a*	0.000	0.000	0.000	1.000
O_1	4b	0.500	0.500	0.500	1.000

Table S6. Crystallographic parameters of 6 wt.% Li-Nb oxide coated sample NMC622PC heated at 1000 °C.

Cell parameters (layered phase, 91 wt.%)<br>Space group: *R-3m*, *a* = *b* = 2.8803, *c* = 14.2585 Å, *α* = *β* = 90°, *γ* = 120°<br>Ni on Li site: 0.1204 ± 0.0018, R_{wp} = 3.99 %<br>Atomic positions					
Name	site	*x*	y	z	occupancy
Li_1	3*a*	0.000	0.000	0.000	0.880
Ni_2	3*a*	0.000	0.000	0.000	0.120
Ni_1	3*b*	0.000	0.000	0.500	0.480
Li_2	3*b*	0.000	0.000	0.500	0.120
Co_1	3*b*	0.000	0.000	0.500	0.200
Mn_1	3*b*	0.000	0.000	0.500	0.200
O_1	6c	0.000	0.000	0.248	1.000
Cell parameters (rock-salt phase, 9 wt.%)<br>Space group: *Fm-3m*, *a* = *b* = *c* = 4.1878 Å, *α* = *β* = *γ* = 90°<br>Atomic positions					
Name	site	*x*	y	z	occupancy
Li_1	4*a*	0.000	0.000	0.000	1.000
Ni_1	4*a*	0.000	0.000	0.000	1.000
Nb_1	4*a*	0.000	0.000	0.000	1.000
O_1	4b	0.500	0.500	0.500	1.000

Table S7. Crystallographic parameters of 5 wt.% Li-Nb oxide coated sample of NMC811SS-800 °C.

Cell parameters (layered phase, 94 wt.%)<br>Space group: *R-3m*, *a* = *b* = 2.8781 Å, *c* = 14.2074 Å, $\alpha = \beta = 90°$, $\gamma = 120°$<br>Ni on Li site: 0.1220 ± 0.0015, R_{wp} = 3.47 %<br>Atomic positions					
Name	site	*x*	y	z	occupancy
Li_1	3*a*	0.000	0.000	0.000	0.878
Ni_2	3*a*	0.000	0.000	0.000	0.122
Ni_1	3*b*	0.000	0.000	0.500	0.678
Li_2	3*b*	0.000	0.000	0.500	0.122
Co_1	3*b*	0.000	0.000	0.500	0.100
Mn_1	3*b*	0.000	0.000	0.500	0.100
O_1	6c	0.000	0.000	0.244	1.000
Cell parameters (rock-salt phase, 6 wt.%)<br>Space group: *Fm-3m*, *a* = *b* = *c* = 4.1910 Å, $\alpha = \beta = \gamma = 90°$<br>Atomic positions					
Name	site	*x*	y	z	occupancy
Li_1	4*a*	0.000	0.000	0.000	1.000
Ni_1	4*a*	0.000	0.000	0.000	1.000
Nb_1	4*a*	0.000	0.000	0.000	1.000
O_1	4b	0.500	0.500	0.500	1.000

Table S8. Crystallographic parameters of 5 wt.% Li-Nb oxide coated sample of NMC811SS-1000 °C.

Cell parameters (layered phase, 95 wt.%)<br>Space group: *R-3m*, $a = b$ = 2.9138 Å, c = 14.2857 Å, $\alpha = \beta$ = 90°, γ = 120°<br>Ni on Li site: 0.3520 ± 0.0019, R_{wp} = 5.66 %<br>Atomic positions					
Name	site	*x*	y	z	occupancy
Li_1	3*a*	0.000	0.000	0.000	0.648
Ni_2	3*a*	0.000	0.000	0.000	0.352
Ni_1	3*b*	0.000	0.000	0.500	0.448
Li_2	3*b*	0.000	0.000	0.500	0.352
Co_1	3*b*	0.000	0.000	0.500	0.100
Mn_1	3*b*	0.000	0.000	0.500	0.100
O_1	6c	0.000	0.000	0.245	1.000
Cell parameters (rock-salt phase, 5 wt.%)<br>Space group: *Fm-3m*, $a = b = c$ = 4.1890 Å, $\alpha = \beta = \gamma$ = 90°<br>Atomic positions					
Name	site	*x*	y	z	occupancy
Li_1	4*a*	0.000	0.000	0.000	1.000
Ni_1	4*a*	0.000	0.000	0.000	1.000
Nb_1	4*a*	0.000	0.000	0.000	1.000
O_1	4b	0.500	0.500	0.500	1.000

Summary of Materials Discussed

This table summarizes active materials, origins, coating (type & loading), coating method, temperature range investigated, detected phases, and reference/source discussed in this manuscript.

**Material**	**Composition**	**Origin / Manufacturer**	**Coating (loading) & Method**	**Temp. Range Investigated**	**Detected Phases (temp info)**	**Reference / Source**
NMC622SC	$LiNi_{0.6}Mn_{0.2}Co_{0.2}O_2$ (single crystal)	MSE Supplies	Li–Nb oxide, 0.5 wt.% (electrochem.), 6 wt.% (XRD); H_2O_2-activated wet coating	350–1000 °C	$LiNbO_3$ (≈400–600 °C), Li_3NbO_4 (≈700–800 °C); rock-salt xLi_3NbO_4-(1-x)$LiMO_2$ observed at 1000 °C	This work
NMC622PC	$LiNi_{0.6}Mn_{0.2}Co_{0.2}O_2$ (polycrystalline)	BASF SE	Li–Nb oxide, 0.5 wt.% / 6 wt.% ; H_2O_2-activated wet coating	350–1000 °C	No crystalline $LiNbO_3$ detected (likely amorphous/nanocrystalline); rock-salt xLi_3NbO_4-(1-x)$LiMO_2$ at high T (800–1000 °C)	This work
NMC811SS (Li_2CO_3)	$LiNi_{0.8}Mn_{0.1}Co_{0.1}O_2$	Self-synthesized (co-precip. precursor + Li_2CO_3, calcined 800 °C)	5 wt.% Li–Nb oxide; H_2O_2-activated wet coating	480–1000 °C	No crystalline $LiNbO_3$ detected; possible Li_3NbO_4/rock-salt at high T	This work
NMC811SS (LiOH)	$LiNi_{0.8}Mn_{0.1}Co_{0.1}O_2$	Self-synthesized (LiOH route, calcined 775 °C)	5 wt.% Li–Nb oxide; H_2O_2-activated wet coating	480–1000 °C	Same: no $LiNbO_3$ observed; rock-salt possible at high T	This work
Ni82	$LiNi_{0.82}Mn_{0.07}Co_{0.11}O_2$	MSE Supplies	5 wt.% Li–Nb oxide; H_2O_2-activated wet coating	350–1000 °C	No crystalline $LiNbO_3$ detected; rock-salt at high T	This work
NMC811X	$LiNi_{0.8}Mn_{0.1}Co_{0.1}O_2$	Ecopro (commercial)	0.7–1.4 wt.% Li–Nb (sol-gel with Nb ethoxide)	400–815 °C	$LiNbO_3$ forms 400–600 °C; Li_3NbO_4 appears ~700–800 °C; rock-salt detected by HR-TEM ~700 °C	Xin et al. (Refs.10,11 in manuscript)
NMC9055	$LiNi_{0.90}Mn_{0.05}Co_{0.05}O_2$	Self-synth. (literature)	Nb-coated/doped (wt.% up to 2.1) via sol-gel; mixed precursors with LiOH	725 °C (single anneal)	Synchrotron peaks assigned to Li_3NbO_4 (questionable); likely formation of Nb-doped NMC + rock-salt at high T	Ref. 20
Ni90533	$LiNi_{0.90}Mn_{0.05}Co_{0.033}O_2$	Self-synth. (literature)	Nb ethoxide + LiOH solid-state mix; heated 775 °C	775 °C	Weak low-angle reflections, unassigned; phase unclear	Ref. 21

NMC811N	$LiNi_{0.8}Mn_{0.1}Co_{0.1}O_2$	Self-synth. (literature)	0.5–5 wt.% Li–Nb via ammonium niobium oxalate (NAmOx) aqueous sol-gel	550 °C	5 wt.% sample shows peaks assigned to $LiNbO_3$ in paper, but assignment is questionable	Ref. 14
NMC622WT	$LiNi_{0.6}Co_{0.2}Mn_{0.2}O_2$	Wujie Technology (commercial)	0.5–2 wt.% (and 20 wt.% ref) Li–Nb via ANO sol-gel	550 °C	Peaks at 23.7°, 42.8°, 62.5° assigned to $LiNbO_3$/Nb_2O_5/Li_3NbO_4 in paper; likely $LiNbO_3$ agglomerates + Li_3NbO_4/rock-salt (assignment debated)	Ref. 15
NMC523N	$LiNi_{0.5}Co_{0.2}Mn_{0.3}O_2$	BASF SE (commercial)	0.5–5 wt.% Li–Nb via ANO aqueous sol-gel	900 °C	Authors report $LiNbO_3$ at 23.7°/32.7°; supporting info Rietveld contradicts → uncertain	Ref. 23
$LiCoO_2$ (commercial)	$LiCoO_2$	Hunan (commercial)	Li–Nb sol-gel (Nb ethoxide + Li_2CO_3)	400–800 °C	$LiNbO_3$ forms 400–600 °C; Li_3NbO_4 forms 600–800 °C; reaction with LCO at higher T	Refs. 26,27
$LiCoO_2$ (self-synth.)	$LiCoO_2$	Self-synth. (solid-state)	Li–Nb sol-gel	400–800 °C	Same sequence: $LiNbO_3$ → Li_3NbO_4 with increasing T	Refs. 26,27
LNMO	$LiNi_{0.5}Mn_{1.5}O_4$ (spinel)	Self-synth. (our earlier work)	2.5 wt.% $LiNbO_3$; H_2O_2-activated coating	up to 800 °C	Crystalline $LiNbO_3$ observed after calcination at high T (800 °C)	Our earlier work (Refs. 3–5 in manuscript)
NMC831	$LiNi_{0.83}Mn_{0.06}Co_{0.11}O_2$	Self-synth. (literature)	Dry coating with Nb_2O_5 → $LiNbO_3$ on CAM; varied Nb content	500 °C	Clear $LiNbO_3$ reflections at low angles after dry coating	Ref. 24
NM82	$LiNi_{0.8}Mn_{0.2}O_2$	Self-synth. (literature)	Dry coating with Nb_2O_5 → $LiNbO_3$	500 °C	Crystalline $LiNbO_3$ observed; increased Nb leads to peak shifts indicating doping	Ref. 13
